# Factors Influencing Preferences of Patients With Rheumatic Diseases Regarding Telehealth Channels for Support With Medication Use: Qualitative Study

**DOI:** 10.2196/45086

**Published:** 2023-07-20

**Authors:** Lex L Haegens, Victor J B Huiskes, Jeffrey van der Ven, Bart J F van den Bemt, Charlotte L Bekker

**Affiliations:** 1 Department of Research and Innovation Sint Maartenskliniek Ubbergen Netherlands; 2 Department of Pharmacy Sint Maartenskliniek Ubbergen Netherlands; 3 Department of Pharmacy Radboud University Medical Center Nijmegen Netherlands

**Keywords:** digital human, drug-related problems, frequently asked questions, interviews, qualitative, rheumatology, support, telehealth, telemedicine

## Abstract

**Background:**

Patients with rheumatic diseases are known to experience drug-related problems at various times during their treatment. As these problems can negatively influence patients’ health, they should be prevented or resolved as soon as possible, for which patients might benefit from additional support. Telehealth has the potential to continuously provide information and offers the possibility to easily contact a health care provider in order to support patients with medication use. Knowledge of factors influencing the patient’s preference for telehealth channels can improve the actual use of telehealth channels.

**Objective:**

This study aims to identify factors that influence the preferences of patients with rheumatic diseases regarding telehealth channels for support with medication use.

**Methods:**

A qualitative study with face-to-face interviews was performed among patients with an inflammatory rheumatic disease in the Netherlands. A total of 4 telehealth channels were used: a frequently asked questions page, a digital human, an app for SMS text messaging with health care providers, and an app for video-calling with health care providers. Using a semistructured interview guide based on domains of the Capability, Opportunity, Motivation, and Behavior (COM-B) model, participants were questioned about (1) their general opinion on the 4 telehealth channels, (2) factors influencing preference for individual telehealth channels, and (3) factors influencing preference for individual telehealth channels in relation to the other available channels. Interviews were recorded, transcribed, and categorically analyzed.

**Results:**

A total of 15 patients were interviewed (female: n=8, 53%; male: n=7, 47%; mean age 55, SD 16.8 years; median treatment duration of 41, IQR 12-106 months). The following 3 categories of factors influencing patient preference regarding telehealth channels were identified: (1) problem-related factors included problems needing a visual check, problems specifically related to the patient, and urgency of the problem; (2) patient-related factors included personal communication preference and patient characteristics; and (3) channel-related factors included familiarity with the telehealth channel, direct communication with a health care provider, methods of searching, and conversation history.

**Conclusions:**

Preference for telehealth channels is influenced by factors related to the problem experienced, the patient experiencing the problem, and telehealth channel characteristics. As the preference for telehealth channels varies between these categories, multiple telehealth channels should be offered to enable patients to tailor the support with their medication use to their needs.

## Introduction

Patients with inflammatory rheumatic diseases often chronically use disease-modifying antirheumatic drugs (DMARDs) to decrease disease-related symptoms and prevent disease progression [1]. However, besides the positive effects of DMARDs, patients also experience multiple drug-related problems (DRPs) at various times during their treatment. On average, patients with rheumatic diseases experience 5 DRPs in an 8-week period, with patients reporting at least 1 new DRP every 2 weeks when followed up for several weeks [2]. For example, more than half of the patients experience side effects from biological DMARDs [3,4], over 90% of patients with rheumatoid arthritis experience at least 1 concern about potential adverse effects of DMARDs [5], and patients experience difficulties with adhering to their medication regimen [6,7]. DRPs have the potential to hamper medication effectiveness and, moreover, jeopardize a patient’s health, resulting in increased morbidity and health care costs [8-10]. It is therefore important to prevent or resolve DRPs as soon as possible.

However, pharmaceutical care management for rheumatic diseases follows standardized follow-up routine visits, which generally entail only a few contact moments between a patient and health care provider annually. Patients only see their health care provider every 3-6 months, while DRPs are even identified every 2 weeks. Thus, DRPs can originate, exist, and potentially worsen for long periods of time before the next routine visit. This discrepancy between the frequency of contact moments and the frequency with which DRPs can occur therefore hinders preventing or resolving DRPs as soon as possible. Additionally, the DRPs experienced are not always discussed during these contact moments [11]. Moreover, in current practice, there exists a mismatch between the patients’ needs for medication-related information and support and the information and support they actually receive. Rheumatology patients receive insufficient information, and the timing of this information is suboptimal [12], and 86% of the patients with systemic sclerosis reported that their need for information is currently not being met [13]. It is therefore assumable that patients can benefit from additional support regarding medication use.

Telehealth refers to the use of digital communication technologies to support long-distance clinical health care, health-related education, health information services, and self-care [14]. Telehealth can provide patients with accessible information and continuous support regarding medication use in between consultations with a health care provider. Previous research shows that telehealth is indeed able to increase access to care and medical information and has the potential to improve patients’ health outcomes and empowerment [15,16]. Furthermore, offering patients telehealth channels is linked to positive effects on health-related knowledge and self-efficacy [17]. As a combination of an aging population and a rise in the number of patients with inflammatory rheumatic diseases limits health care providers’ time and resources, telehealth may provide opportunities to lighten the burden of these challenges [18].

Given the potential of telehealth, the implementation of blended care (ie, offering telehealth services in addition to traditional face-to-face care) might improve the pharmaceutical care of patients with inflammatory rheumatic diseases. However, despite patients’ willingness to adopt telehealth, actual use of telehealth among patients with rheumatic diseases remains low [19]. Knowledge of patient preferences for telehealth channels can increase actual use and potentially help reduce DRPs. This study therefore aims to identify factors that influence the preferences of patients with rheumatic diseases regarding telehealth channels for support with medication use.

## Methods

### Study Design and Setting

A qualitative study with individual face-to-face interviews was performed at the rheumatology department of the Sint Maartenskliniek, a hospital in the Netherlands specializing in the treatment of conditions relating to posture and movement, between May and June 2021.

### Participants

Adult patients (18 years of age or older) diagnosed with an inflammatory rheumatic disease and receiving a DMARD from the outpatient pharmacy as part of their treatment were eligible to participate. Additionally, participants had to be able to communicate in Dutch and live in the area of the hospital to avoid the burden of traveling for the face-to-face interviews. Eligible patients with a scheduled (in-person or telephone) consultation with their rheumatologist during the study period were selected from the hospital information system and approached for participation by phone. Interested patients received written information and an informed consent form by mail or e-mail. When patients agreed to participate, the interviews were scheduled directly after their visit to the rheumatologist for convenience reasons or at a suitable time for participants who had a telephone consultation with the rheumatologist. To ensure a diverse sample, patients were recruited by means of purposive sampling based on age, gender, treatment duration, and type of consultation (in-person or telephone). Recruitment of new participants was continued until data saturation was reached (ie, until no new categories emerged from the data) [20]. Written informed consent was obtained before the interview started.

### Interview Guide

An interview guide was constructed based on the Capability, Opportunity, Motivation, and Behavior (COM-B) model [21]. This model states that in order for a behavior to occur, a person needs the capability, opportunity, and motivation to engage in the behavior. Capability concerns an individual’s psychological and physical capacity to engage in the activity concerned (eg, possessing necessary knowledge and skills). Opportunity concerns all factors outside an individual, both social and physical, which make the behavior possible or prompt it. Motivation concerns all psychological processes, both automatic and reflective, that direct behavior. Choosing a telehealth channel for medication support can be viewed as a behavior. As it is therefore subject to the COM-B model, this model was chosen as the theoretical basis for the interview guide to identify factors that influence this behavior. The interview guide consisted of questions based on each of these domains. It was tested on 3 nonpatients, refined twice, and can be found in Multimedia Appendix 1.

### Interviews

Interviews were conducted by 1 researcher (JV), who was assisted by the first author (LLH) during the first 3 interviews. No relationship was established between participants and interviewers prior to the study. During each interview, participants were asked to use 4 telehealth channels: a frequently asked questions page (FAQ), a digital human (which is a humanlike virtual being that can recreate natural human interactions using artificial intelligence [22] and enable users to verbally ask questions and receive spoken answers back), an app for SMS text messaging with a health care provider, and an app for video-calling with a health care provider.

First, the 4 telehealth channels were introduced to the participants by the interviewer. Then, to familiarize themselves with each channel, participants were instructed to find an answer to a predetermined medication-related question using all 4 channels (“Am I allowed to drink alcohol while using methotrexate?”), during which they were asked to communicate their thoughts and opinions to the researcher by thinking aloud. This predetermined question was chosen to ensure all telehealth channels provided the participant with an answer to enable participants to practice and experience using the channel. After finding the answer to the predetermined question with each channel, participants were asked about factors that influenced their preference for each telehealth channel using the questions included in the interview guide (Multimedia Appendix 1). Lastly, participants were presented with several varying predefined medication-related scenarios (Multimedia Appendix 1) and asked to rank the 4 telehealth channels on use preference, after which they were asked about factors determining the order of preferred channels. Lastly, participants were asked about the possible benefits and disadvantages of telehealth channels as an addition to usual care.

### Data Analysis

Interviews were transcribed verbatim, after which transcripts were analyzed deductively using categorical analysis. First, during open coding, relevant quotes were labeled. Second, during axial coding, open codes relating to the same were grouped. Third, the main categories were constructed from axial codes. All analyses were performed in Atlas.ti 9 [23]. Analysis started with the open coding of 1 transcript by 2 researchers (LLH and CLB) independently. Discrepancies in coding were discussed and resolved. Subsequently, LLH coded the remaining interviews, of which 3 were reviewed by a third researcher (VJBH). Discrepancies were again discussed and resolved. Recurring codes were grouped into factors by LLH, which were reviewed by BJFB, CLB, and VJBH. From these factors, categories were constructed independently by CLB and LLH, which, through discussion, resulted in a prefinal categorization. Lastly, the prefinal categorization was discussed and finetuned by BJFB, CLB, LLH, and VJBH until consensus on the final categories was reached, that is, all authors agreed that the constructed categories represented the data.

### Ethical Considerations

This study was performed in accordance with the Declaration of Helsinki. The Medical Research Ethics Committee of Arnhem-Nijmegen, the Netherlands, waived official ethical approval (case number 2021-8181) and assessed this study as not being subject to the Medical Research Involving Human Subjects Act. All participants gave written informed consent before the interview started.

## Results

### Overview

A total of 15 participants were interviewed. Data saturation was reached after 12 interviews (ie, no new categories emerged from the data), after which 3 more interviews were conducted. There were 8 (53%) female participants; the mean age was 55 (SD 16.8) years; the median treatment duration was 41 (IQR 12-106) months; and 8 (53%) participants had a face-to-face consultation. The most common diagnoses were rheumatoid arthritis (n=7, 47%), psoriatic arthritis (n=3, 20%), polymyalgia rheumatica (n=2, 13%), and axial spondylarthritis (n=2, 13%). The mean duration of the interviews was 50 (SD 9) minutes. All participants were in possession of at least one mobile device (eg, smartphone, tablet, or laptop) or desktop.

### General Experience

Overall, participants were positive regarding the use of telehealth channels to communicate, acquire information, and receive support with their medication use. Participants had no previous experience with the specific channels used in this study, except for the FAQ page.

**Figure 1:**
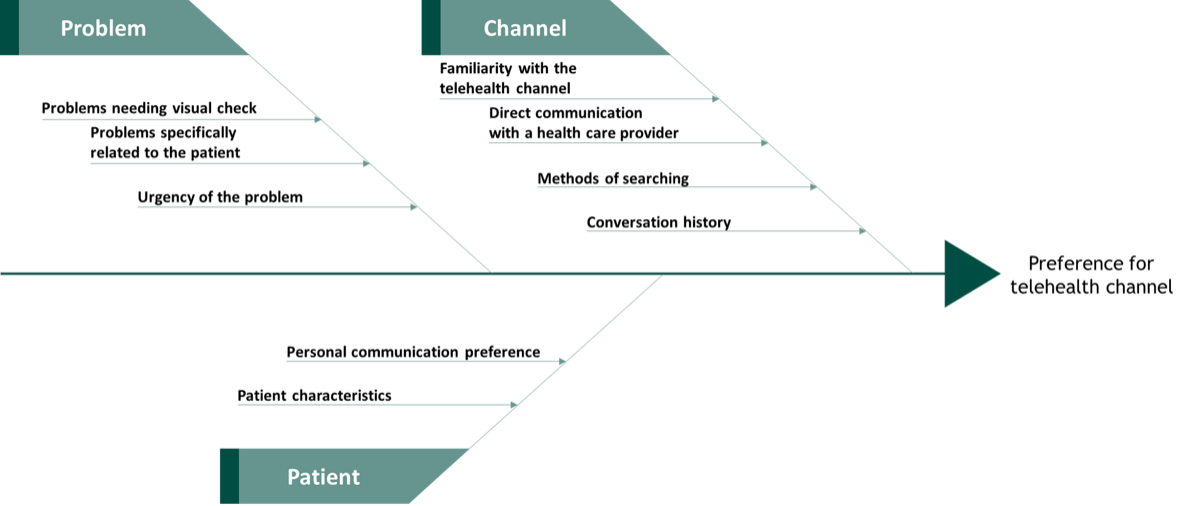
Categories and corresponding factors that influence patient preference for telehealth channels.

The following 3 categories of factors influencing patient preference for telehealth channels were identified: factors related to the problem experienced, factors related to the patient experiencing the problem, and factors related to the telehealth channel (Figure 1).

### Factors Related to the Medication-Related Problem

#### Problems Needing Visual Check

Participants reported that problems requiring a visual check, such as a rash or swollen joint, prompted a need for visual communication with a health care provider. As such, this influenced the choice for a certain telehealth channel, with text messaging and video calling being the only channels with the possibility to include imagery (ie, photo or video).

When talking to my doctor on the telephone, we cannot see each other. For example, he or she cannot see that my finger is swollen. While I think it might be inflamed. If I am able to show the doctor what I mean, then he or she can assess if action should be taken or not.

Female, 56 years

#### Problem Specifically Related to the Patient

Participants mentioned that their preference is influenced by the extent to which a problem or needed answer is specific to their individual situation. Participants would rather discuss problems specifically related to themselves directly with a health care provider, while they mostly preferred the FAQ and the digital human for more general problems, such as a need for general information, as human interaction was not necessary to get such questions answered.

In response to the question in which a patient considers stopping using medication due to side effects:

Then I would want personal contact. I think I would call first and ask for a video call. Because I think addressing the problem through the FAQ is too risky. The doctor knows better who I am than the general questions.

Female, 56 years

Within problems specific to a patient, participants indicated that they sometimes have difficulty discussing certain sensitive topics directly with a health care provider, such as nonadherence to medication or recreational drug use, and therefore preferred to use channels that do not involve direct or face-to-face contact with a health care provider.

Maybe you are uncomfortable asking your doctor face-to-face regarding recreational drug use, so you would rather do it anonymously using the digital human or the chat.

Female, 34 years

#### Urgency of the Problem

The urgency of the problem influenced the preference for telehealth channels. In general, patients indicated that the speed at which an answer is received is important when choosing a channel for urgent problems. SMS text messaging was preferred over video calling for urgent problems, as there is no need to book an appointment in advance with a health care provider for text messaging using the messaging platform used in this study.

If you are in great pain, you want a response as soon as possible.

Male, 51 years

### Factors Related to the Patient Experiencing the Problem

#### Personal Communication Preference

The 2 methods of communication offered by the telehealth channels were either verbally (spoken questions and spoken answers) or textually (typed questions and written answers). Patients varied in their preference for telehealth channels based on the method of communication of each channel.

In response to the question why the participant disliked the digital human in general:

That is a matter of personal preference, I think. You have to speak out loud what you intend to say. I’m more of a typer than a speaker in that respect.

Male, 30 years

#### Patient Characteristics

Participants indicated that their preference for telehealth channels was influenced by various personal characteristics. For instance, age could influence this preference, as participants believed younger patients to have more technical skills and may therefore be more capable of downloading and installing certain apps, whereas older patients might struggle. Therefore, channels available on a website, such as the FAQ, could be more preferable for older patients.

I think for older people, it's going to take more effort before the app is installed on their smartphone.

Male, 19 years

Furthermore, participants indicated that their therapy phase had an influence on their preference for telehealth channels. Starting a new therapy was believed to lead to a higher need for information related to medication use in between consultations and therefore a preference for telehealth channels that could serve as a source of information, such as the FAQ or the digital human.

I've been using methotrexate for a long time. But in the beginning when I started using it, I had a lot of questions. What are the side effects, do I have to keep using medication for the rest of my life, and so on. You have all those kinds of questions then.

Male, 71 years

### Factors Related to the Telehealth Channel

#### Familiarity With the Telehealth Channel

Participants mentioned that some channels were comparable with existing apps, for instance, SMS text messaging and WhatsApp or video calling and FaceTime. They indicated that this familiarity improved the ease of use of such channels when provided by the hospital and increased their preference for such channels as compared to channels with which they were less familiar.

I am used to chatting so I do find that very convenient I must say. In time you receive an answer which is the same when I message someone via WhatsApp.

Female, 70 years

#### Direct Communication With a Health Care Provider

Participants indicated that the possibility of direct, one-on-one interaction with a health care provider also influences their preference for telehealth channels, regardless of the problem they experience. This included both face-to-face interactions using video calling as well as chatting directly with a health care provider. Some participants indicated that being able to see the health care provider was more satisfactory, as they rely partly on nonverbal communication in these situations. Other participants indicated that this type of interaction was not a prerequisite for addressing a medication-related question or problem.

You're directly interacting with someone, with a person. I think that is satisfying. More satisfying than if you were to read it on a website.

Male, 19 years

#### Methods of Searching

The method of searching for information using a channel influenced the preference for telehealth channels. Some participants indicated that they preferred actively searching for an answer themselves in a database of information as a way to gain more information, but most participants preferred to passively receive a direct answer to their question without having to search for it, as this was seen as more convenient. This was illustrated by a preference for the digital human over the FAQ, for example:

I can just look it up myself. I personally find that easy.

Female, 49 years

So instead of conducting a search myself, I would ask the digital human. It can do the preliminary work.

Female, 34 years

#### Conversation History

Participants found the fact that some channels offered the possibility to reread answers and functioned as a reference source for questions asked in the past to be a positive influence on their preference for use. Being able to re-read complex answers after, for example, SMS text messaging with a health care provider was seen as an added value compared to phone or video calling.

Well, with a phone call you can’t have another look at the answers. I think it is convenient that the answer is in writing, so you can check to see whether you understood it correctly. So that is an advantage of this feature.

Male, 71 years

### Requirements for Adequate Use of Telehealth

In addition to factors influencing preference for individual telehealth channels, participants also named several preconditions for adequate use of telehealth channels. Participants deem clear instructions and the possibility to practice with specific telehealth channels as beneficial for future use. Furthermore, cooperation with and support from their health care providers, as well as support from others (eg, family or friends), are necessary for patients to be able to optimally use telehealth channels. Additionally, participants indicated that the trustworthiness of the information and its source are important for good use of telehealth channels. As telehealth channels and their contents are from a trusted source (ie, the hospital) when compared to information from other sources (eg, on the internet), this can help reassure patients when they are uncertain. Lastly, privacy and data security must be guaranteed to facilitate safe and effective use of telehealth channels, as this concerns information about the patient’s health and health care.

### Benefits of Telehealth in Addition to Usual Care

Participants reported factors related to the benefits that telehealth channels have when available in addition to usual care. Participants indicated that the availability of telehealth channels can improve access to care, as they feel they can more easily and quickly access hospital information or contact health care providers themselves. Furthermore, telehealth enables patients to access information and health care regardless of place and time, which leads to patients feeling more at ease.

Telehealth channels can also prevent physical strain, as patients are able to act on problems from their homes. Problems can be solved without waiting for the next consultation, which in some cases renders face-to-face routine check-ups superfluous.

If this can replace a consultation, that would be great. It would be much easier if it were possible to ask questions in between consultations without having to go to the hospital. I normally have to come here by bus, which costs a lot of time.

Female, 49 years

Lastly, participants indicated that they were hesitant to ask certain questions as they felt they would be bothering overburdened health care providers, so they were then more inclined to use the telehealth options for these questions. Thus, telehealth channels can shift care consumption. On the one hand, this can result in more questions being asked, but on the other hand, this can also aid in preventing a worsening of problems that would normally result in increased care consumption as problems can be addressed at an earlier stage.

I can ask questions without the feeling of being an annoyance.

Male, 73 years

## Discussion

### Principal Results

This qualitative study showed that preference for telehealth channels is influenced by factors related to 3 main categories: the problem that is experienced, the patient that experiences the problem, and the characteristics of the telehealth channel. Additionally, requirements for adequate use of telehealth channels and factors related to the benefits of telehealth channels in combination with usual care were identified. Overall, participants were positive regarding telehealth channels.

### Comparison With Prior Work

Previous studies have researched the views of rheumatology patients regarding telehealth in the context of planned consultations. Some studies show patients’ preference for telehealth over face-to-face consultations [24-26], while others show the opposite [27]. Additionally, health care providers in rheumatology show low satisfaction with telehealth as alternative to planned face-to-face consultations due to technical difficulties and a lack of physical examination, among others [28]. Although these studies provide relevant context for the use of telehealth channels in rheumatology in general, our research differs from these studies in the fact that the aim of this study was to assess factors influencing patients’ preferences for different telehealth channels to contact their health care providers or access information in between their regular contacts when they are at home.

Our results are in line with previous research on factors influencing the preference for telehealth channels. A systematic review by Crossnohere et al [29] found that preference for telehealth channels is dependent on the type of problem that is communicated, with more sensitive, urgent, or complex health concerns preferably being communicated directly with a health care provider. Alexander et al [30] found similar results, with the proportion of patients in an Australian hospital willing to communicate digitally instead of face-to-face being influenced by the level of concern regarding experienced symptoms, concluding that less concerned patients were more willing to use telehealth to communicate. In line with this study, several other studies also identified factors related to the patient that influence the choice for telehealth channels, such as age, gender, and education [29,31]. The identified channel-related factors in this study are also reported in other studies, showing that trustworthiness of sources and familiarization with channels influence patients’ willingness to use such channels [32,33]. So, this study confirms factors that influence patients’ preferences for individual telehealth channels that have been found in other studies. The added value of this study is that these factors also influence patients’ preferences regarding the choice between several telehealth channels instead of only the preference for telehealth over face-to-face contact or vice versa.

### Implications

In current practice, there exists a mismatch between patients’ needs for medication related information and support and the information and support they actually receive. Furthermore, two-thirds of rheumatology patients believed telehealth was helpful for their health, yet only 4% of patients actually used medical apps [19]. As this study showed that preference for telehealth channels is dependent on factors related to the problem, the patient, and characteristics of the channel, a tailored or customizable approach would optimally support patients with their medication use rather than a one-size-fits-all approach. Especially since our findings indicate that the preference for telehealth channels might differ within individual patients based on their situation. The factors found in this study can be used to determine what kinds of channels and features could be offered to patients to increase actual use. Future research could, for example, look at possibilities for anonymously chatting or mailing directly with a health care provider to enable patients to communicate problems regarding sensitive topics, or using artificial intelligence to create minutes of conversations in channels that currently do not keep these records. This way, patients can choose the most suitable channel in each situation by themselves. As previous research indicated that awareness of the advantages of telehealth in rheumatology is low [32], it is important to promote telehealth channels to patients and guide them on how to effectively use such channels so they can make a more educated choice for the most suitable channel. In the future, it is important to measure the effects of telehealth channels in terms of actual usage, health-related outcomes, and cost-effectiveness to assess if the offered range of telehealth channels reach their full potential.

### Limitations

Some limitations should be acknowledged. First, the generalizability of this study’s results might be limited. Participants were recruited from a single center, which is the largest specialized rheumatology center in the Netherlands; thus, patients’ demographics and indications might differ from the general rheumatology population. Furthermore, as telehealth was assessed as an addition to usual care, participants’ preferences might differ from those of other centers as the level of usual care is different. Nonetheless, we believe that the data are generalizable for problems experienced with medication use for chronic diseases, as treatment for these diseases is highly standardized in the Netherlands. Secondly, although participants were purposively sampled based on age, gender, and treatment experience, self-selection bias might have led to a study population that is more interested in telehealth than the average population of patients with rheumatic diseases. Thirdly, as the data collection consisted of face-to-face interviews, there is the possibility that participants gave socially desirable answers during the interviews. To limit the impact on the internal validity of the collected data, questions were formulated as neutrally and openly as possible, and patients were assured that interviews had no influence on their treatment. Furthermore, there was no previous relationship between the interviewer and the participants. Lastly, the outcomes of this study might be influenced by the COVID-19 pandemic that took place during data collection. As a result of social distancing measures in the Netherlands, standard care partly shifted from face-to-face to video consultations. This possible previous experience with one of the telehealth channels used in this study could have influenced what factors influence a patient’s preference for telehealth channels.

### Conclusions

This study found that patients’ preferences for telehealth channels are influenced by factors related to the problem experienced, the patient experiencing the problem, and the telehealth channel used to address this problem. As preferences for telehealth channels vary between these categories among patients, multiple telehealth channels should be offered to patients to improve actual use by enabling patients to tailor the support to their needs.

## Appendix

Multimedia Appendix 1 Translation of the original interview guide.

## Abbreviations

COM-B: Capability, Opportunity, Motivation, and Behavior
DMARD: disease-modifying antirheumatic drugs
DRP: drug-related problems
FAQ: frequently asked questions
