# Supplementary material for: Factors Influencing Preferences of Patients With Rheumatic Diseases Regarding Telehealth Channels for Support With Medication Use: Qualitative Study
Source: JMIR Form Res. 2023 Jul 20;7:e45086. doi: 10.2196/45086 (PMC10401190; doi:10.2196/45086)
Supplement: Multimedia Appendix 1 [file formative_v7i1e45086_app1.docx]

**Translation of the original interview guide**

General questions

1. Do you possess a smartphone, laptop, tablet or computer?
   - How often do you use these devices?
   - How easy do you find using these devices?

Introduction of the telehealth channels

- Please think aloud during the introduction of each telehealth channel.

Using the telehealth channels

1. Were you already familiar with this telehealth channel?
   - If yes, what was your experience with this channel?
2. What is your opinion on this telehealth channel?
   - What do you like about it, and why?
   - What do you dislike about it, and why?
3. What do you think of the ease of use of this telehealth channel?
   - What do you think makes it easy to use?
   - What could be improved to make it easier to use?
4. Do you think you would be capable to use this telehealth channel on your own?
   - What skills should one possess to effectively use this telehealth channel?
5. If this telehealth channel would be available, how likely are you use this telehealth channel for support with medication use?
   - If likely, what factors cause this?
   - If unlikely, what would you need to become likely to use the telehealth channel?

Ranking telehealth channels

1. Please explain what factors made you rank the telehealth channels as you did.
   - What positives of the most preferred telehealth channel(s) do you see when compared to the other channels??
   - What negatives of the least preferred telehealth channel(s) do you see when compared to the other channels?
2. You did not choose [specific telehealth channel], please explain what factors made you not choose this channel.
   - What factors could cause you to (re)consider this telehealth channel?

General opinion on telehealth channels as addition to usual care

1. What do you think of offering these telehealth channels as addition to usual care?
   - What positives do you see?
   - What negatives do you see?

**Example questions used for the ranking exercise**

- “I just had to vomit, does my medication still work?”
- “I am experiencing side effects from my medication, can I stop taking them?”
- “The power of my refrigerator went out, does my medication still work?”
- “I forgot to take my last dose of medication, can I still take them?”
- “What long-term illnesses or side-effects can I expect from these medications, like liver or kidney problems, or cancer?”
- “What happens when treatment is not effective and there are no other treatment options left?”
- “Can I combine this medication with other medications I am already taking?”
- “I have the desire to have children, is there anything I should take into account regarding my current medication use?”
- “Why do I have to get my blood checked regularly since taking this medication?”
- “I would like to know more about the working mechanism of this medication.”
- “What are the known side-effects of this medication?”
